# Response of Iranian lizards to future climate change by poleward expansion, southern contraction, and elevation shifts

**DOI:** 10.1038/s41598-022-06330-4

**Published:** 2022-02-11

**Authors:** Somaye Vaissi

**Affiliations:** Department of Biology, Faculty of Science, Razi University, Baghabrisham, Kermanshah, Iran

**Keywords:** Climate sciences, Climate change

## Abstract

This study explores the relationships between recent Iranian lizard species distributions and the observed climate, as well as potential future distributions of species. For this purpose, an ensemble of seven algorithms was used to forecast the distributions of 30 species for the recent and future (2070) based on the averages of 14 global climate models under optimistic (RCP2.6) and pessimistic (RCP8.5) scenarios. Annual precipitation (n = 16) and annual mean temperature (n = 7) were identified as the most important variables in determining the distribution of 76.66% (23 out of 30) of the species. The consensus model predicts that the ranges of 83.33% of species (n = 25) have the potential to expand poleward at higher latitudes while preserving the majority of their recent distributions (except for four species). Furthermore, the ranges of the remaining species (n = 5) will be preserved at higher latitudes. However, they (n = 22) may contract slightly (n = 13) or excessively (n = 9) in the south of their distribution range at lower latitudes. These results indicate that species (N = 19) situated in mountainous areas such as the Zagros, Alborz, and Kopet Dagh may move or maintain their range at higher elevations as a result of future climate change. Finally, this study suggests that 30% of species (n = 9) may be threatened by future climate change and that they should be prioritized in conservation efforts.

## Introduction

Climate change poses a serious threat to the world's biodiversity^1^. It affects many aspects of populations, including distribution, behaviour, physiology, phenology, and the tendency for local extinction^2–6^. As a result of future climate change, many species that cannot adapt will need to shift poleward in latitude, lower in water depth, higher in altitude, or to refugial areas that may be outside their current or previous native ranges^7^. According to predictive models, if climate change continues uncontrolled, 37% of global species will be extinct by 2050^8^. Therefore, one of the most challenging tasks facing conservation biologists is assessing species responses to climate change^9–13^. Especially for species with limited dispersal ability, they may have difficulty colonizing suitable environments due to their specific ecological needs^14,15^. For example, it is predicted that 98% and 59% of European and South African reptiles, respectively, will become extinct or contract their ranges if they are unable to migrate, these numbers are decreased to 35% and 0% if they can migrate^9,14^. Hence, the first step in mitigating the impacts on biodiversity is identifying the most vulnerable species or groups of species that are likely to be impacted by changing climatic circumstances^16^.

Terrestrial ectotherms, particularly reptiles, have declined and been extirpated in many parts of the world, and climate change is one of the primary causal agents proposed to explain these decreases^17–20^. This is since their ecology and biology are intricately related to climate, particularly temperature fluctuations in the environment^21,22^. Climate change, in particular, poses a serious threat to several populations of lizards around the world, and these populations are expected to decline over the next century^18^. This is especially true for tropical lizards, which are already nearing their physiological optimum^23^. Physiological stress, lower performance, and increased disease susceptibility result from body temperatures that are greater than optimal, eventually leading to population decreases and extinction^15,18^. Temperature increases of 1.1 to 6.4 °C until 2100, for example, would increase ectotherm metabolic rates by 10–75%^24^. Increased metabolic rates combined with decreased foraging time may have a negative impact on reproduction and, as a result, population growth rates^24,25^. The interaction of these effects creates disturbances in metapopulation dynamics and population, which may eventually lead to distribution changes^26^.

Despite the reported negative consequences of climate change, it is predicted that warmer temperatures may be expected to benefit some species by expanding ranges into currently unoccupied areas^27–31^. Increases in mean temperature, on the other hand, may have a positive influence on mid-latitude lizards by prolonging the growing season and in the frequency in which species experience temperatures close to their optimal ones^32^. However, it is important to note that the risks of higher heat stress levels and durations during the summer, particularly in adults, may counteract the benefits^33–35^. On the other hand, evidence shows that some species remain in large portions of their ranges despite a climatic change or to extend into new niches. For example, Hickling et al. (2006) found that increased temperatures have not affected the distribution of *Natrix natrix* and *Lacerta agilis* in the United Kingdom^36^. Moreno-Rueda et al. (2011) suggested that climate change may be shifting the latitudinal distributions of Spanish reptiles^37^. Sinervo et al. (2017) reported range expansions in four of seven *Sceloporus* lizards with high body temperatures (T_b_) in Mexico^38^. They found climate change may improve local conditions for lizards with higher T_b_ that are restricted by cold at their high altitudes^38^. Carvalho et al. (2010) used species distribution projections to assess the impact of climate change on 37 Iberian Peninsula herptiles^26^. They found that the distributions of 46% of species will decrease, while the distributions of 28% may expand.

**Figure 1:**
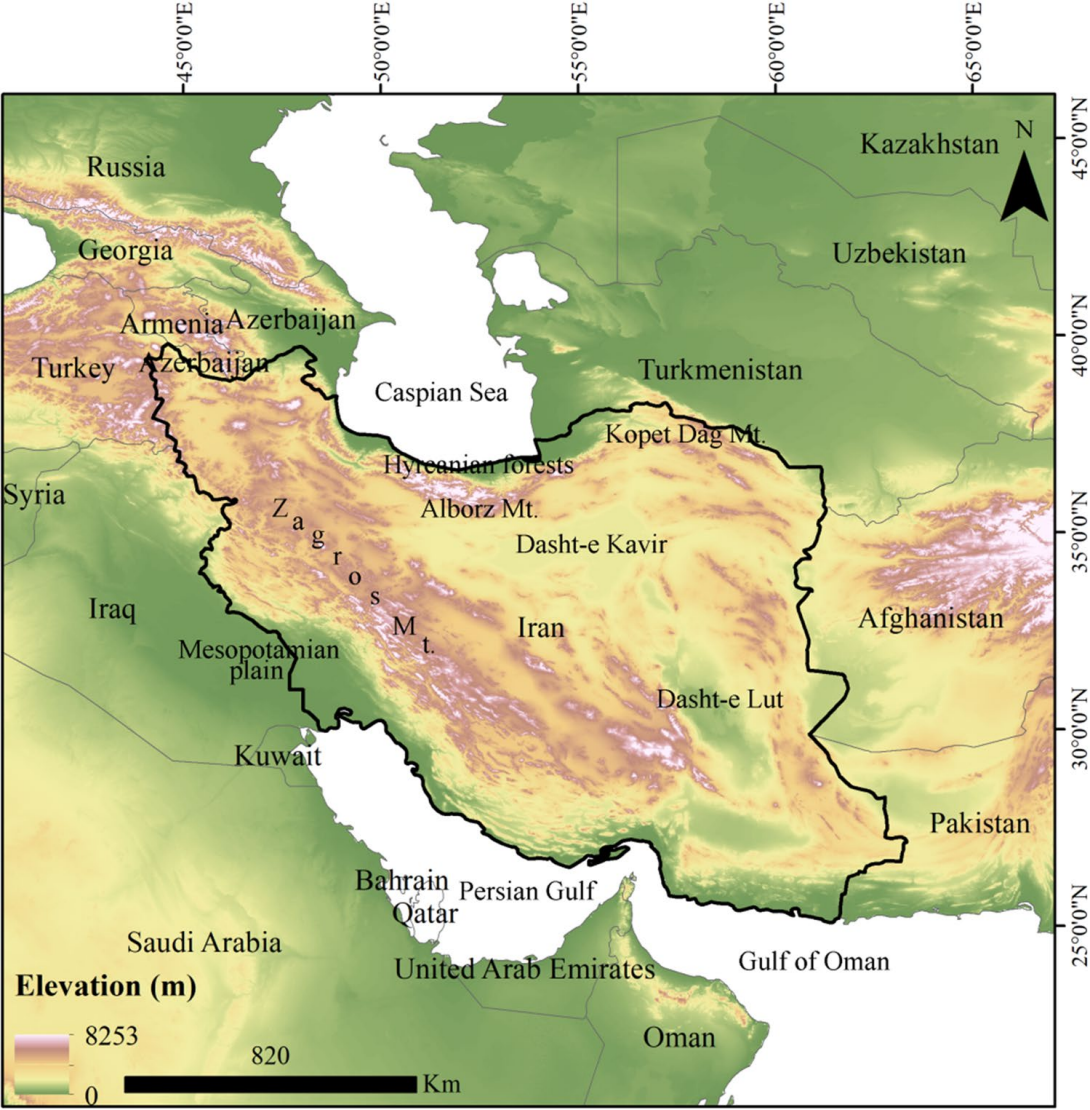
Study area. Iran. Map was generated using ArcMap (v 10.8) (https://desktop.arcgis.com).

Iran (Fig. 1) is one of the richest countries in southwest Asia in terms of biodiversity that have 171 species of lizards, with more than 62 (36.25%) of them being endemic to the country^39^. However, Iran's future climate is expected to be unpleasant, with consecutive periods of extreme humidity and dryness throughout the country^40,41^. According to projections from the Intergovernmental Panel on Climate Change (IPCC), based on the assumption that greenhouse gas emissions will continue to rise throughout the twenty-first century, Iran might experience a temperature rise of 1.5 to 4.5 °C by 2100 ^42^. According to Daneshvar et al. (2019), the temperature will rise between 1.12 and 7.87°C^43^. Vaghefi et al. (2017) anticipated this number to be between 1.1 and 2.75°C^40^. For this purpose, this study used 30 species from 22 genera of lizards throughout the country to estimate how their distribution might shift in the face of future climate change. In particular, ensemble species distribution modelling using seven algorithms, was used to determine (a) what climatic factors might be driving projections of gain or loss in suitable habitats for these species in the future (2070)? (b) what proportion of these lizards species are projected to gain and lose their suitable habitats? and (c) which species should be prioritized in conservation efforts?

## Results

**Figure 2:**
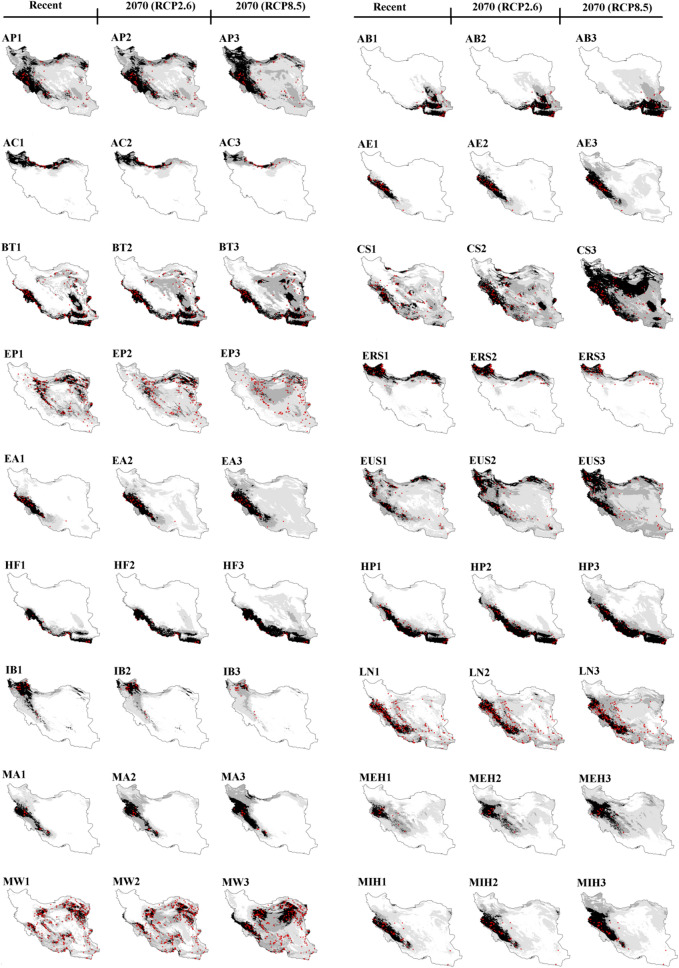

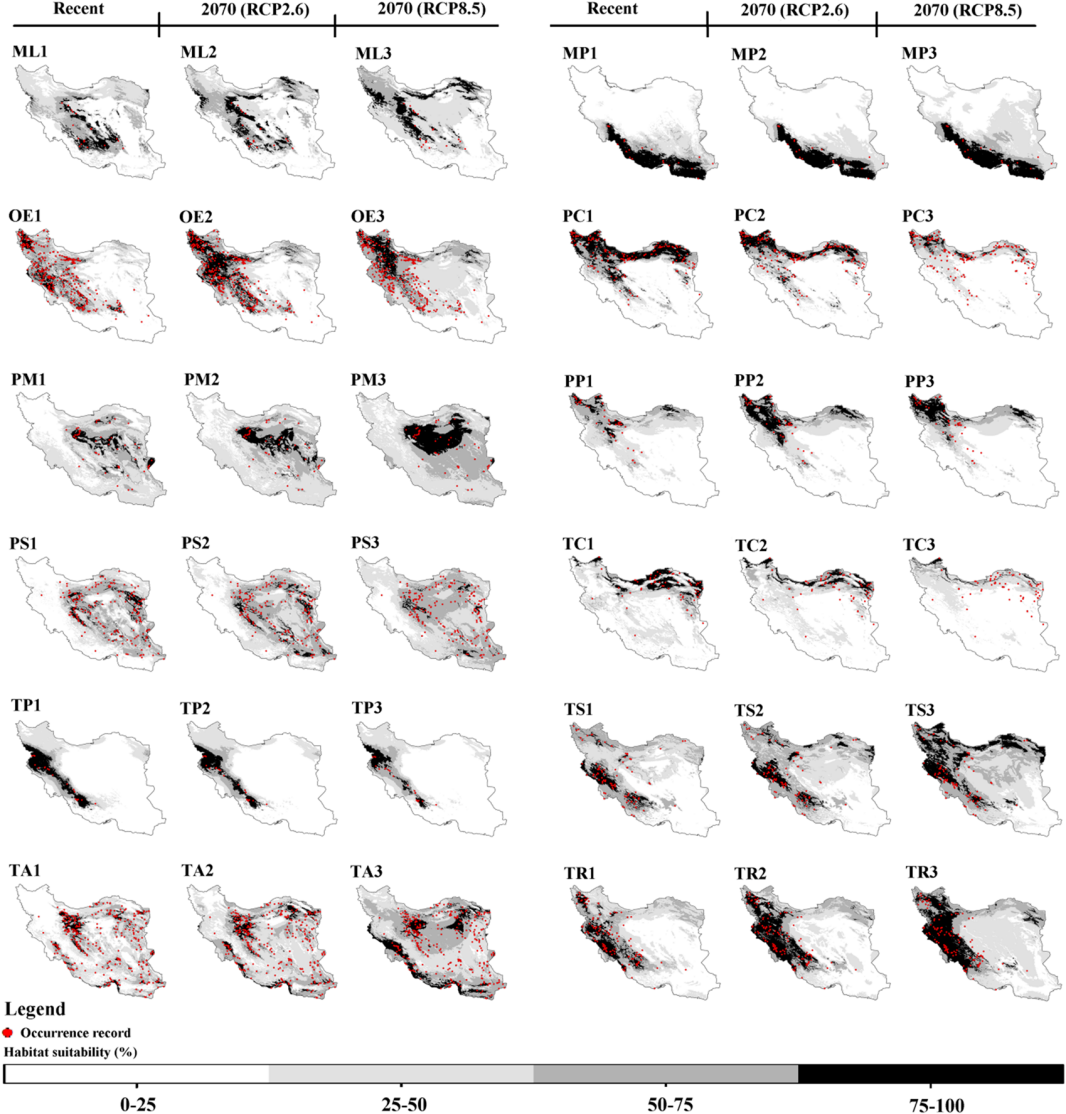
Recent and future (2070) habitat suitability (%) for 30 lizard species based on the consensus model under optimistic (RCP2.6) and pessimistic (RCP8.5) scenarios in Iran. (AP) *Ablepharus pannonicus*; (AB) *Acanthodactylus blanfordii*; (AC) *Anguis colchica*; (AE) *Asaccus elisae,* (BT*) Bunopus tuberculatus*; (CS) *Cyrtopodion scabrum*; (EP) *Eremias persica*; (ERS) *Eremias strauchi;* (EA) *Eublepharis angramainyu*; (EUS) *Eumeces schneideri*; (HF) *Hemidactylus flaviviridis*; (HP) *Hemidactylus persicus*; (IB) *Iranolacerta brandtii*; (LN) *Laudakia nupta*; (MA) *Mediodactylus aspratilis*; *Mediodactylus heterocercum* (MEH); (MW) *Mesalina watsonana*; (MIH) *Microgecko helenae*; (ML) *Microgecko latifi*; (MP) *Microgecko persicus*; (OE) *Ophisops elegans*; (PC) *Paralaudakia caucasia*; (PM) *Phrynocephalus maculatus*; (PP) *Phrynocephalus persicus*; (PS) *Phrynocephalus scutellatus*; (TC) *Tenuidactylus caspius*; (TP) *Timon princeps*; (TS) *Trachylepis septemtaeniata*; (TA) *Trapelus agilis*; (TR) *Trapelus ruderatus*. Maps were generated using ArcMap (v 10.8) (https://desktop.arcgis.com).

**Figure 3:**
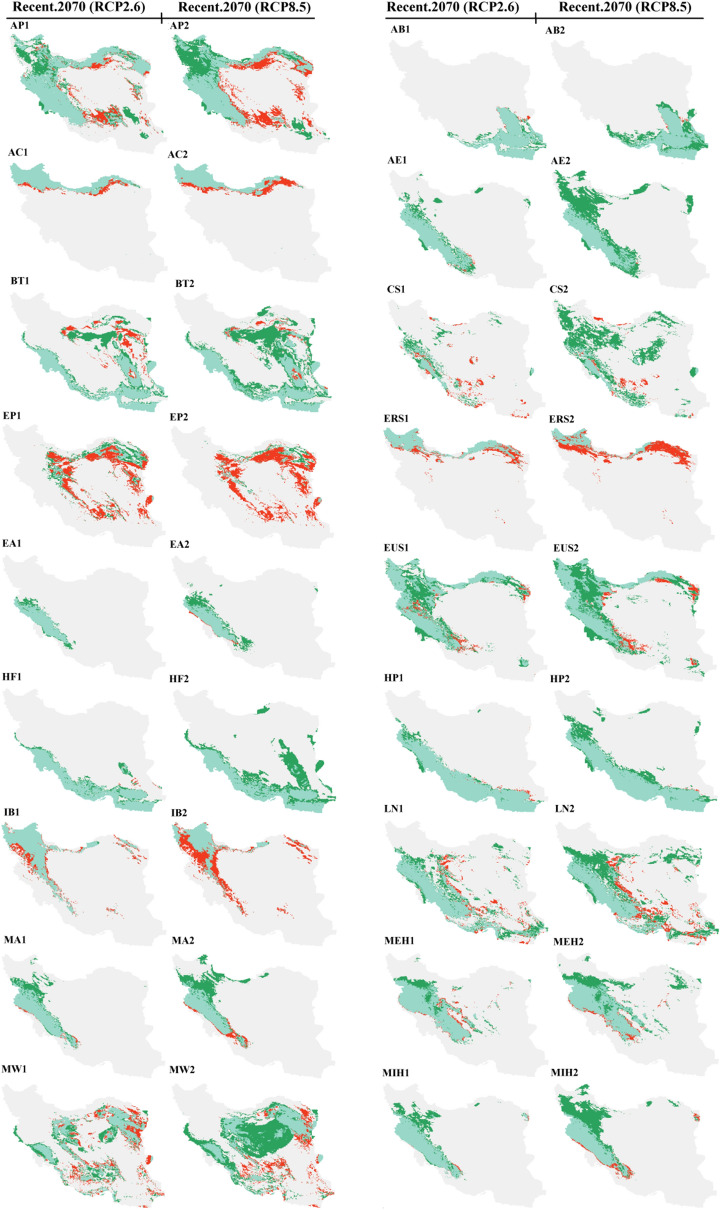

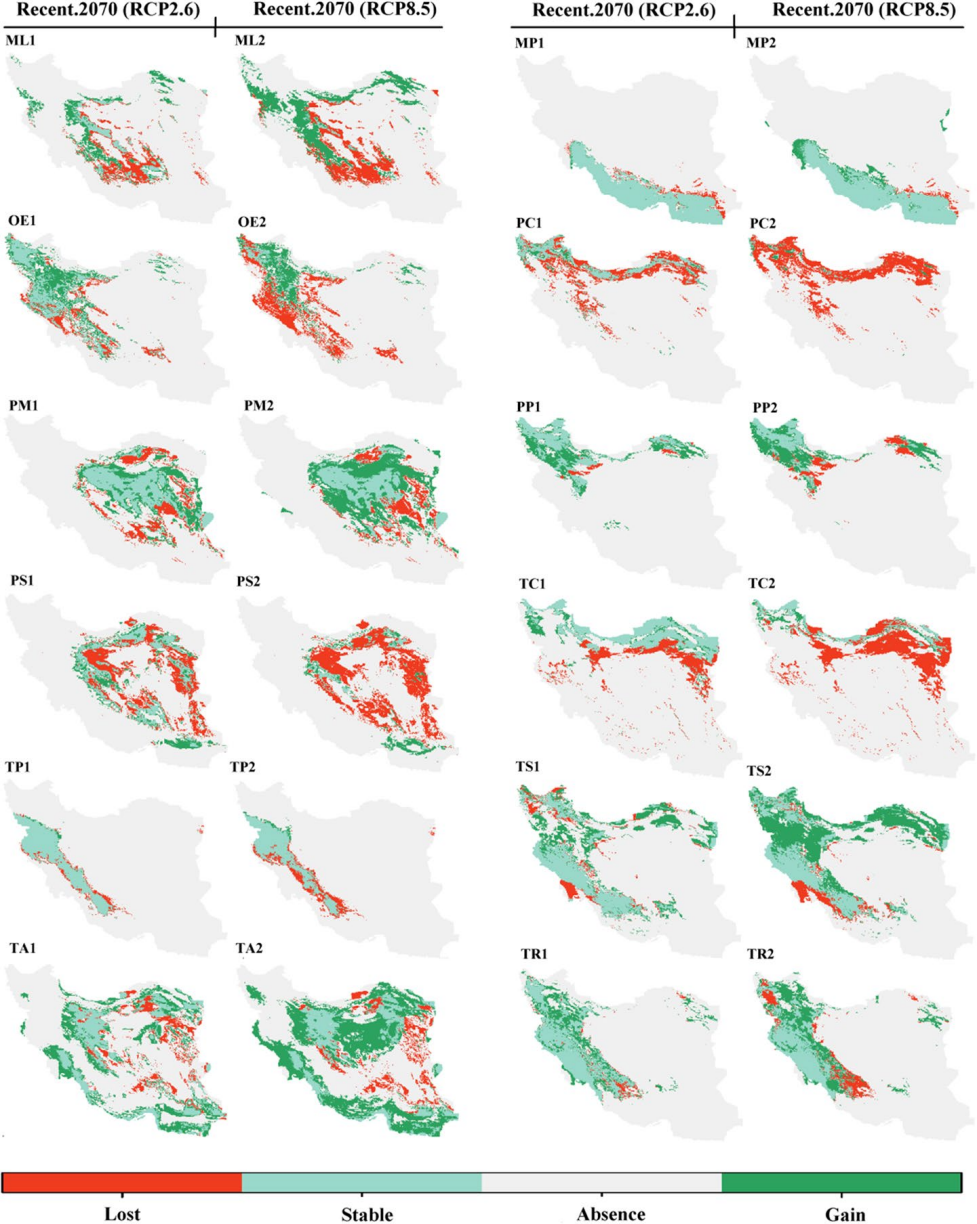
Species range change of 30 species of lizards in recently suitable habitats (gain/loss) by 2070 under optimistic (RCP2.6) and pessimistic (RCP8.5) scenarios in Iran. (AP) *Ablepharus pannonicus*; (AB) *Acanthodactylus blanfordii*; (AC) *Anguis colchica*; (AE) *Asaccus elisae,* (BT*) Bunopus tuberculatus*; (CS) *Cyrtopodion scabrum*; (EP) *Eremias persica*; (ERS) *Eremias strauchi;* (EA) *Eublepharis angramainyu*; (EUS) *Eumeces schneideri*; (HF) *Hemidactylus flaviviridis*; (HP) *Hemidactylus persicus*; (IB) *Iranolacerta brandtii*; (LN) *Laudakia nupta*; (MA) *Mediodactylus aspratilis*; *Mediodactylus heterocercum* (MEH); (MW) *Mesalina watsonana*; (MIH) *Microgecko helenae*; (ML) *Microgecko latifi*; (MP) *Microgecko persicus*; (OE) *Ophisops elegans*; (PC) *Paralaudakia caucasia*; (PM) *Phrynocephalus maculatus*; (PP) *Phrynocephalus persicus*; (PS) *Phrynocephalus scutellatus*; (TC) *Tenuidactylus caspius*; (TP) *Timon princeps*; (TS) *Trachylepis septemtaeniata*; (TA) *Trapelus agilis*; (TR) *Trapelus ruderatus*. Maps were generated using R (v 4.2.0) (https://cran.r-project.org).

The ensemble models exhibited high quality, with TSS, AUC, and KAPPA values ranging from 0.76 to 1 (Table 1). The map of suitable habitats based on elevation and bioclimatic variables for recent and future (2070) climate conditions under RCP2.6 and RCP8.5 scenarios for 30 species of lizards that are distributed in Iran is shown in Fig. 2. The mean of variable importance (%) as estimated by the algorithms for the 30 species of lizards are provided in Table 2. For 16 species BIO12, for seven species BIO1, for three species BIO15, for two species BIO5 and one species elevation were found to be the most important variables affecting the distribution of species. Table 3 and Fig. 3 illustrate the range shift of 30 species of lizards in recently suitable habitats (gain/loss) by 2070 under the RCP2.6 and RCP8.5 scenarios.

**Table 1:** Lizard species list, codes, conservation status under IUCN criteria, diurnal (D) or nocturnal (N) activity, number of occurrence records (N), and ensemble model quality.

No.	Species	Code	IUCN status	Diurnal (D)/nocturnal (N)	Occurrence records (N)	Ensemble model quality		
						TSS	AUC	KAPPA
1	*Ablepharus pannonicus*	AP	Not Listed	D	90	0.82	0.96	0.79
2	*Acanthodactylus blanfordii*	AB	Not Listed	D	64	0.86	0.97	0.78
3	*Anguis colchica*	AC	Not Listed	D	34	0.93	0.99	0.86
4	*Asaccus elisae*	AE	Least Concern	N	49	0.93	0.99	0.91
5	*Bunopus tuberculatus*	BT	Least Concern	N	156	0.81	0.97	0.82
6	*Cyrtopodion scabrum*	CS	Least Concern	D	125	0.85	0.95	0.78
7	*Eremias persica*	EP	Not Listed	D	193	0.92	0.97	0.91
8	*Eremias strauchi*	ERS	Least Concern	D	75	0.87	0.98	0.89
9	*Eublepharis angramainyu*	EA	Data Deficient	N	39	0.91	0.99	0.91
10	*Eumeces schneideri*	EUS	Least Concern	D	88	0.80	0.96	0.81
11	*Hemidactylus flaviviridis*	HF	Not Listed	D	35	0.90	0.98	0.81
12	*Hemidactylus persicus*	HP	Not Listed	D	62	0.80	0.97	0.79
13	*Iranolacerta brandtii*	IB	Data Deficient	D	32	0.93	0.99	0.89
14	*Laudakia nupta*	LN	Not Listed	D	255	0.85	0.98	0.87
15	*Mediodactylus aspratilis* *	MA	Data Deficient	D/N	20	0.93	0.98	0.93
16	*Mediodactylus heterocercum*	MEH	Least Concern	D	20	0.93	0.97	0.87
17	*Mesalina watsonana*	MW	Not Listed	D	398	0.84	0.98	0.86
18	*Microgecko helenae* *	MIH	Data Deficient	D	54	0.82	0.97	0.82
19	*Microgecko latifi* *	ML	Least Concern	D/N	22	0.89	0.98	0.85
20	*Microgecko persicus*	MP	Not Listed	D	25	0.84	0.97	0.83
21	*Ophisops elegans*	OE	Least Concern	D	409	0.94	0.98	0.95
22	*Paralaudakia caucasia*	PC	Least Concern	D	198	0.80	0.97	0.85
23	*Phrynocephalus maculatus*	PM	Not Listed	D	58	0.84	0.97	0.82
24	*Phrynocephalus persicus*	PP	Vulnerable	D	75	0.83	0.97	0.84
25	*Phrynocephalus scutellatus*	PS	Not Listed	D	227	0.98	1.00	0.98
26	*Tenuidactylus caspius*	TC	Least Concern	D	86	0.78	0.96	0.76
27	*Timon princeps* *	TP	Least Concern	D/N	24	0.93	1.00	0.91
28	*Trachylepis septemtaeniata*	TS	Least Concern	D	99	0.87	0.97	0.83
29	*Trapelus agilis*	TA	Not Listed	D	387	0.95	0.98	0.94
30	*Trapelus ruderatus*	TR	Least Concern	D	144	0.83	0.96	0.87

*Endemic to Iran.

**Table 2:** Mean of variable importance (%) by the algorithms for the 30 lizard species in Iran.

No.	Species	BIO1	BIO4	BIO5	BIO12	BIO14	BIO15	Elevation
1	*Ablepharus pannonicus*	12.86	10.42	10.65	49.76	1.45	3.85	10.98
2	*Acanthodactylus blanfordii*	30.09	20.52	7.23	25.33	1.13	9.34	6.32
3	*Anguis colchica*	9.77	3.18	22.01	5.88	29.48	27.75	1.88
4	*Asaccus elisae*	6.32	4.60	31.90	41.35	4.23	6.12	5.46
5	*Bunopus tuberculatus*	28.22	4.14	10.01	5.74	3.36	5.02	43.46
6	*Cyrtopodion scabrum*	20.89	8.92	30.02	20.68	2.42	7.35	9.68
7	*Eremias persica*	38.04	7.87	12.37	23.67	2.64	8.37	7.00
8	*Eremias strauchi*	11.03	3.30	11.97	15.92	22.59	28.18	6.98
9	*Eublepharis angramainyu*	9.35	4.20	31.76	39.80	2.55	6.62	5.68
10	*Eumeces schneideri*	4.19	12.61	11.27	55.41	5.04	6.89	4.56
11	*Hemidactylus flaviviridis*	29.24	3.55	5.19	6.75	16.39	23.88	14.97
12	*Hemidactylus persicus*	11.09	16.35	8.90	24.95	2.03	31.00	5.64
13	*Iranolacerta brandtii*	14.32	5.70	6.46	36.85	17.90	8.71	10.03
14	*Laudakia nupta*	8.88	9.03	21.43	36.79	5.49	11.95	6.35
15	*Mediodactylus aspratilis*	3.16	6.10	12.93	61.48	5.57	6.52	5.21
16	*Mediodactylus heterocercum*	25.54	7.12	19.68	18.57	4.10	9.42	15.55
17	*Mesalina watsonana*	19.33	12.94	11.61	36.88	5.41	7.61	6.19
18	*Microgecko helenae*	6.63	4.18	13.40	56.24	8.11	6.50	4.92
19	*Microgecko latifi*	26.31	12.88	26.91	9.09	5.59	19.20	16.66
20	*Microgecko persicus*	14.11	7.03	5.42	9.80	4.50	50.85	8.25
21	*Ophisops elegans*	14.01	17.69	10.25	39.50	1.08	6.04	11.40
22	*Paralaudakia caucasia*	32.99	7.82	25.07	9.12	3.52	17.83	3.61
23	*Phrynocephalus maculatus*	14.03	15.33	17.42	36.73	1.19	6.41	8.85
24	*Phrynocephalus persicus*	18.96	6.81	13.61	22.31	12.01	21.77	4.49
25	*Phrynocephalus scutellatus*	22.49	9.62	6.65	44.04	2.60	8.74	5.82
26	*Tenuidactylus caspius*	30.36	4.94	18.15	8.21	0.83	9.10	28.37
27	*Timon princeps*	8.14	5.79	4.21	53.03	4.15	14.41	10.23
28	*Trachylepis septemtaeniata*	6.44	7.40	16.90	57.67	1.56	3.53	6.47
29	*Trapelus agilis*	24.36	21.08	12.61	20.93	2.62	11.83	6.53
30	*Trapelus ruderatus*	10.19	5.84	12.02	58.52	2.71	5.64	5.04

Annual mean temperature (BIO1); temperature seasonality (BIO4); the max temperature of the warmest month (BIO5); annual precipitation (BIO12); precipitation of driest month (BIO14); and precipitation seasonality (BIO15).

**Table 3:** Species range change (gain/loss) of 30 lizard species in recently suitable habitats by 2070 under optimistic (RCP2.6) and pessimistic (RCP8.5) scenarios in Iran. Species that are threatened by future climate change have also been identified.

No.	Species	RCP2.6			RCP8.5			
		Lost	Gain	Tendency	Lost	Gain	Tendency	Conservation attention
1	*Ablepharus pannonicus*	17.59	31.32	Expansion	30.98	37.60	Expansion	−
2	*Acanthodactylus blanfordii*	1.59	17.03	Expansion	1.09	49.80	Expansion	−
3	*Anguis colchica* ^1^	14.17	1.73	Contraction	27.83	0.34	Contraction	−
4	*Asaccus elisae*	2.00	45.20	Expansion	0.20	152.49	Expansion	−
5	*Bunopus tuberculatus*	17.19	48.37	Expansion	8.42	93.04	Expansion	−
6	*Cyrtopodion scabrum*	40.10	69.33	Expansion	27.87	319.38	Expansion	−
7	*Eremias persica*	74.37	33.75	Contraction	90.19	15.08	Contraction	+
8	*Eremias strauchi*	25.28	0.14	Contraction	61.09	0.00	Contraction	+
9	*Eublepharis angramainyu*	0.10	30.78	Expansion	4.61	68.58	Expansion	−
10	*Eumeces schneideri*	9.03	53.45	Expansion	14.04	70.92	Expansion	−
11	*Hemidactylus flaviviridis*	1.27	20.03	Expansion	0.00	83.46	Expansion	−
12	*Hemidactylus persicus*	1.74	11.21	Expansion	0.41	34.24	Expansion	−
13	*Iranolacerta brandtii*	20.13	0.46	Contraction	47.76	0.12	Contraction	+
14	*Laudakia nupta*	16.20	43.13	Expansion	24.58	73.37	Expansion	−
15	*Mediodactylus aspratilis*	3.95	43.83	Expansion	14.12	76.70	Expansion	−
16	*Mediodactylus heterocercum*	7.79	25.07	Expansion	7.92	48.43	Expansion	−
17	*Mesalina watsonana*	34.06	40.18	Expansion	30.01	103.58	Expansion	−
18	*Microgecko helenae*	1.85	47.78	Expansion	11.15	95.07	Expansion	−
19	*Microgecko latifi* ^2^	58.74	60.15	Expansion	92.84	122.23	Expansion	+
20	*Microgecko persicus*	9.20	2.47	Contraction	5.23	18.31	Expansion	−
21	*Ophisops elegans*	33.20	66.52	Expansion	78.89	51.85	Contraction	+
22	*Paralaudakia caucasia*	59.32	9.25	Contraction	94.67	4.03	Contraction	+
23	*Phrynocephalus maculatus*	37.58	57.18	Expansion	35.19	94.16	Expansion	−
24	*Phrynocephalus persicus*	10.91	87.11	Expansion	37.32	90.96	Expansion	−
25	*Phrynocephalus scutellatus*	59.74	28.89	Contraction	88.10	17.13	Contraction	+
26	*Tenuidactylus caspius*	39.90	16.07	Contraction	79.30	10.26	Contraction	+
27	*Timon princeps*	14.55	2.63	Contraction	29.83	4.15	Contraction	+
28	*Trachylepis septemtaeniata*	20.19	58.73	Expansion	20.77	107.96	Expansion	−
29	*Trapelus agilis*	31.87	72.15	Expansion	34.17	143.79	Expansion	−
30	*Trapelus ruderatus*	8.21	52.36	Expansion	25.74	67.24	Expansion	−

^1^Although habitat loss is greater than new habitat gain, the majority of existing habitats will remain under the influence of the future climate, see Figs. 2 and 3.

^2^Although new habitat gain is greater than habitat loss, the majority of existing habitats will be lost under the influence of the future climate, see Figs. 2 and 3.

Future climate change will affect 30 Iranian lizards in different ways, some by expanding their ranges, some by contracting their ranges, and others by remaining relatively unaffected (especially in habitats with 75–100% suitability) (Tables 3 and 4). The species of AP, AB, AE, BT, CS, EP, EA, EUS, HF, HP, LN, MA, MEH, MW, MIH, ML, MP, OE, PM, PP, PS, TP, TS, TA, and TR expanded northward at higher latitudes while preserving the majority of their recent distribution (except for EP, OE, PS, and TP) (Figs. 2 and 3). Furthermore, the ranges of the AC, ERS, IB, PC, and TC will be preserved at higher latitudes. However, they may contract slightly (AC, CS, EUS, LN, MA, MEH, MW, MIH, PP, TP, TS, TA, and TR) or excessively (AP, EP, ERS, IB, ML, OE, PC, PS, and TC) in the south of their distribution range at lower latitudes (Figs. 2 and 3). The species of AP, AE, CS, EP, EA, EUS, HF, LN, MA, MEH, MW, MIH, ML, OE, PM, PS, TC, TS, and TR may move or maintain their range at higher elevations as a result of future climate change. The following are details of 30 species' responses to climate change (Figs. 2 and 3).

**Table 4:** The habitat area suitability (%) and its coverage (%) within the protected areas (PAs) network for the response of 30 lizard species to future (2070) climate change under a pessimistic (RCP8.5) scenario compared to recent climatic conditions. + denotes a positive impact; − denotes a negative impact.

No.	Species	Suitability classes (%)	Habitat area suitability (%)/its coverage (%) within PAs		Potential impacts
			Recent	2070 (RCP8.5)	
1	*Ablepharus pannonicus*	0–25	16.93/1.66	1.21/0.04	−/−
		25–50	42.76/3.27	51.60/4.45	+/+
		50–75	29.19/2.65	26.43/2.51	−/−
		75–100	11.12/0.39	20.76/0.97	+/+
2	*Acanthodactylus blanfordii*	0–25	76.03/5.47	52.51/3.68	−/−
		25–50	8.36/0.75	24.34/2.08	+/+
		50–75	7.22/1.00	11.95/1.70	+/+
		75–100	8.41/0.75	11.22/0.50	+/−
3	*Anguis colchica*	0–25	81.34/6.53	85.08/6.75	+/+
		25–50	6.07/0.38	5.24/0.41	−/ +
		50–75	4.59/0.22	6.96/0.62	+/+
		75–100	8.02/0.84	2.74/0.19	−/−
4	*Asaccus elisae*	0–25	85.17/7.33	45.52/3.94	−/−
		25–50	7.61/0.49	34.12/3.02	+/+
		50–75	2.34/0.07	12.10/0.77	+/+
		75–100	4.90/0.08	8.27/0.24	+/+
5	*Bunopus tuberculatus*	0–25	51.85/3.71	25.63/1.40	−/−
		25–50	17.46/1.38	17.14/1.40	−/ +
		50–75	15.85/1.36	38.02/3.87	+/+
		75–100	14.85/1.52	19.23/1.30	+/−
6	*Cyrtopodion scabrum*	0–25	31.33/2.16	1.80/0.13	−/−
		25–50	35.80/2.80	10.65/0.95	−/−
		50–75	24.11/2.43	41.75/2.92	+/+
		75–100	8.77/0.57	45.80/3.96	+/+
7	*Eremias persica*	0–25	39.96/2.77	21.20/1.23	−/−
		25–50	31.87/2.80	49.02/3.75	+/+
		50–75	20.16/1.60	27.58/2.79	+/+
		75–100	8.03/0.80	2.22/0.20	−/−
8	*Eremias strauchi*	0–25	71.39/5.78	79.05/6.41	+/+
		25–50	10.91/0.95	10.86/0.75	−/−
		50–75	7.11/0.44	6.07/0.52	−/ +
		75–100	10.61/0.79	4.04/0.30	−/−
9	*Eublepharis angramainyu*	0–25	75.47/6.68	13.63/1.10	−/−
		25–50	14.56/0.84	61.40/5.61	+/+
		50–75	4.43/0.36	15.60/0.95	+/+
		75–100	5.55/0.09	9.39/0.32	+/+
10	*Eumeces schneideri*	0–25	16.29/1.72	0.16/0.01	−/−
		25–50	51.19/4.20	36.04/4.14	−/−
		50–75	23.60/1.53	46.90/2.93	+/+
		75–100	8.93/0.53	16.92/0.89	+/+
11	*Hemidactylus flaviviridis*	0–25	77.10/5.91	50.03/3.27	−/−
		25–50	8.44/1.33	26.79/2.87	+/+
		50–75	6.76/0.17	8.57/1.12	+/+
		75–100	7.71/0.56	14.61/0.71	+/+
12	*Hemidactylus persicus*	0–25	64.19/5.76	36.04/3.01	−/−
		25–50	13.33/1.24	34.49/3.45	+/+
		50–75	9.11/0.42	11.69/0.83	+/+
		75–100	13.40/0.56	17.79/0.69	+/+
13	*Iranolacerta brandtii*	0–25	70.42/6.34	77.02/6.68	+/+
		25–50	13.84/0.69	14.63/0.94	+/+
		50–75	8.51/0.61	7.20/0.32	−/−
		75–100	7.24/0.33	1.15/0.03	−/−
14	*Laudakia nupta*	0–25	42.87/4.42	14.39/1.83	−/−
		25–50	33.34/2.25	43.20/4.09	+/+
		50–75	15.06/0.90	32.83/1.74	+/+
		75–100	8.73/0.41	9.59/0.30	+/−
15	*Mediodactylus aspratilis*	0–25	74.49/6.91	66.62/6.51	−/−
		25–50	13.93/0.74	11.47/0.40	−/−
		50–75	6.13/0.13	12.40/0.79	+/+
		75–100	5.46/0.19	9.52/0.27	+/+
16	*Mediodactylus heterocercum*	0–25	54.77/5.20	28.40/2.52	−/−
		25–50	28.75/2.06	45.20/4.21	+/+
		50–75	12.32/0.56	17.23/0.87	+/+
		75–100	4.18/0.16	9.19/0.37	+/+
17	*Mesalina watsonana*	0–25	41.61/3.64	15.20/0.72	−/−
		25–50	27.39/2.50	34.60/2.92	+/+
		50–75	22.73/1.37	33.45/2.91	+/+
		75–100	8.27/0.47	16.76/1.42	+/+
18	*Microgecko helenae*	0–25	59.83/6.09	44.34/4.86	−/−
		25–50	29.11/1.54	34.34/2.11	+/+
		50–75	3.90/0.13	10.77/0.71	+/+
		75–100	7.06/0.20	10.57/0.29	+/+
19	*Microgecko latifi*	0–25	42.72/4.06	35.68/3.25	−/−
		25–50	30.37/4.06	38.92/3.06	+/−
		50–75	19.83/2.18	14.87/1.04	−/−
		75–100	7.08/1.12	10.54/0.63	+/−
20	*Microgecko persicus*	0–25	57.67/0.62	39.14/2.95	−/ +
		25–50	18.92/4.48	35.40/3.57	+/−
		50–75	8.16/2.12	9.31/0.76	+/−
		75–100	14.88/0.78	15.93/0.67	+/−
21	*Ophisops elegans*	0–25	50.74/4.66	28.07/1.58	−/−
		25–50	21.30/1.72	37.33/4.22	+/+
		50–75	21.49/1.10	25.69/1.62	+/+
		75–100	6.47/0.49	8.92/0.55	+/+
22	*Paralaudakia caucasia*	0–25	57.12/5.28	72.16/6.15	+/−
		25–50	10.94/0.60	17.58/0.79	+/+
		50–75	13.73/0.89	7.57/0.73	−/−
		75–100	18.22/1.20	2.71/0.30	−/−
23	*Phrynocephalus maculatus*	0–25	25.90/1.26	6.67/0.30	−/−
		25–50	47.27/3.45	47.56/2.01	+/−
		50–75	21.11/2.30	31.49/4.20	+/+
		75–100	5.73/0.95	14.29/1.46	+/+
24	*Phrynocephalus persicus*	0–25	58.99/4.98	64.41/5.13	+/+
		25–50	24.04/1.97	17.26/1.58	−/−
		50–75	13.37/0.88	11.20/0.71	−/−
		75–100	3.63/0.14	7.15/0.54	+/+
25	*Phrynocephalus scutellatus*	0–25	40.15/2.57	13.22/0.41	−/−
		25–50	26.80/2.64	44.71/3.99	+/+
		50–75	28.11/2.43	39.50/3.40	+/+
		75–100	4.96/0.32	2.58/0.16	−/−
26	*Tenuidactylus caspius*	0–25	54.82/4.51	74.98/6.24	+/+
		25–50	28.33/2.09	19.37/1.25	−/−
		50–75	9.19/0.65	4.41/0.31	−/−
		75–100	7.67/0.72	1.24/0.17	−/−
27	*Timon princeps*	0–25	67.10/6.27	71.34/6.73	+/+
		25–50	18.27/1.20	16.43/0.84	−/−
		50–75	6.58/0.20	8.04/0.25	+/+
		75–100	7.93/0.29	4.21/0.16	−/−
28	*Trachylepis septemtaeniata*	0–25	40.91/3.62	18.10/0.64	−/−
		25–50	27.71/2.23	31.94/3.70	+/+
		50–75	24.58/1.88	31.06/2.59	+/+
		75–100	6.82/0.24	18.91/1.05	+/+
29	*Trapelus agilis*	0–25	35.13/3.44	2.69/0.21	−/−
		25–50	38.59/2.61	39.64/3.12	+/+
		50–75	20.86/1.41	44.54/3.85	+/+
		75–100	5.42/0.52	13.14/0.79	+/+
30	*Trapelus ruderatus*	0–25	40.94/3.81	14.80/0.55	−/−
		25–50	29.81/2.36	45.69/5.22	+/+
		50–75	19.80/1.30	19.55/1.53	−/ +
		75–100	9.46/0.49	19.98/0.66	+/+

### *A. pannonicus* (AP)

BIO12 (49.76%) and BIO1 (12.86%) are the two important variables affecting the distribution of Asian Snake-eyed Skink, respectively (Table 2). The habitat suitability map for recent climate conditions shows that the west and southwest, along with the Zagros, Alborz, and Kopet Dagh ranges, as well as a portion of the northwest and northeast, have 50 to 100% suitability. Habitats with 75 to 100% suitability are concentrated in the middle and southern Zagros, a narrow strip of Alborz, and a small section of northeastern Kopet Dagh, as well as the northwestern and northeastern parts of the country (Fig. 2 AP1). Based on future climate change (especially RCP8.5), suitable habitats (especially 75–100% suitability), in addition to maintaining the recent range, will expand to higher latitudes in the north and northwest. While the eastern and southern margins of the distribution range will contract in the face of future climate change (Fig. 2 AP2 and AP3 and Fig. 3 AP1 and AP2). Expansion to higher elevations for habitats with 75–100% suitability is also apparent in the Zagros, Alborz, and Kopet Dagh mountains (Fig. 2 AP2 and AP3). Based on the consensus model under RCP2.6 and RCP8.5, habitat loss was estimated at 17.59% and 30.98%, and new habitat gain at 31.32% and 37.60%, respectively (Table 3).

### *A. blanfordii* (AB)

BIO1 (30.09%) and BIO12 (25.33%) are the two important variables affecting the distribution of Blanford’s Fringe-toed Lizard, respectively (Table 2). The habitat suitability map for recent climate conditions shows that the southeast of Iran has 50 to 100% (especially 75–100%) suitability (Fig. 2 AB1). Based on future climate change (especially RCP8.5), suitable habitats, in addition to maintaining the recent range, will expand to higher latitudes (Fig. 2 AB2 and AB3 and Fig. 3 AB1 and AB2). Based on the consensus model under RCP2.6 and RCP8.5, habitat loss was estimated at 1.59% and 1.09%, and new habitat gain at 17.03% and 49.80%, respectively (Table 3).

### *A. colchica* (AC)

BIO14 (29.48%) and BIO15 (27.75%) are the two important variables affecting the distribution of Colchican Slow Worm, respectively (Table 2). The habitat suitability map for recent climate conditions shows that the north along the Caspian coast, the central and eastwards up of Kopet Dagh, and the northwest of Iran have 50 to 100% (especially 75–100%) suitability (Fig. 2 AC1). Based on future climate change (especially RCP8.5), suitable habitats (especially 75–100% suitability) will be maintained in higher latitudes. While the southern margins of the distribution range will decrease as a result of future climate change (Fig. 2 AC2 and AC3 and Fig. 3 AC1 and AC2). Based on the consensus model under RCP2.6 and RCP8.5, habitat loss was estimated at 14.17% and 27.83%, and new habitat gain at 1.73% and 0.34%, respectively (Table 3).

### *A. elisae* (AE)

BIO12 (41.35%) and BIO5 (31.90%) are the two important variables affecting the distribution of Elisa’s Leaf-toed Gecko, respectively (Table 2). The habitat suitability map for recent climate conditions shows that the west and southwest, along with the Zagros, have 50 to 100% (especially 75–100%) suitability (Fig. 2 AE1). Based on future climate change (especially RCP8.5), suitable habitats (especially 50–75% suitability), in addition to maintaining the recent range, will expand to higher latitudes in the north and northwest of Iran (Fig. 2 AE2 and AE3 and Fig. 3 AE1 and AE2). Expansion to higher elevations for habitats with 75–100% suitability is also apparent in the Zagros mountains (Fig. 2 AE2 and AE3). Based on the consensus model under RCP2.6 and RCP8.5, habitat loss was estimated at 2.00% and 0.20%, and new habitat gain at 45.20% and 152.49%, respectively (Table 3).

### *B. tuberculatus* (BT)

Elevation (43.46%) and BIO1 (28.22%) are the two important variables affecting the distribution of Tuberculated Desert Gecko, respectively (Table 2). The habitat suitability map for recent climate conditions shows that the Mesopotamian plain, the Iranian Plateau from south of the Kopet Dagh to the coastal areas around the Strait of Hormuz and the Gulf of Oman have 50 to 100% suitability. Habitats with 75 to 100% suitability are concentrated in the Mesopotamian plain, coastal areas around the Strait of Hormuz and the Gulf of Oman to the southeast of Iran (Fig. 2 BT1). Based on future climate change (especially RCP8.5), suitable habitats (especially 50–75% suitability), in addition to maintaining the recent range, will expand to higher latitudes in the southeast, east, and east to the center of Iran (Fig. 2 BT2 and BT3 and Fig. 3 BT1 and BT2). Based on the consensus model under RCP2.6 and RCP8.5, habitat loss was estimated at 17.19% and 8.42% and new habitat gain at 48.37% and 93.04%, respectively (Table 3).

### *C. scabrum* (CS)

BIO5 (30.02%) and BIO1 (20.89%) are the two important variables affecting the distribution of Rough-tail Bent-toed gecko, respectively (Table 2). The habitat suitability map for recent climate conditions shows that the Hyrcanian forests, Mesopotamian plain, the west and southwest, along with the Zagros, south and southwest regions, and the central Plateau, have 50 to 100% suitability (Fig. 2 CS1). Based on future climate change (especially RCP8.5), suitable habitats (especially 75–100% suitability), in addition to maintaining the recent range, will expand to higher latitudes in the northwest, north, central, and northeast of Iran. A slight decrease may also occur in the south of the range (Fig. 2 CS2 and CS3 and Fig. 3 CS1 and CS2). Expansion to higher elevations for habitats with 75–100% suitability is apparent in the Zagros and Kopet Dagh mountains (Fig. 2 CS2 and CS3). Based on the consensus model under RCP2.6 and RCP8.5, habitat loss was estimated at 40.10% and 27.87% and new habitat gain at 69.33% and 319.38%, respectively (Table 3).

### *E. persica* (EP)

BIO1 (38.04%) and BIO12 (23.67%) are the two important variables affecting the distribution of the Persian Desert Lacerta, respectively (Table 2). The habitat suitability map for recent climate conditions shows that the eastern parts of the Zagros mountains, the whole central plateau (except the Dasht-e Kavir and the Dasht-e Lut deserts), and the southern parts of the Alborz mountains have 50 to 100% suitability (Fig. 2 EP1). Based on future climate change (especially RCP8.5), suitable habitats will be shifted to higher latitudes in the higher elevations of the Alborz mountains and northeast of Iran (Fig. 2 EP2 and EP3 and Fig. 3 EP1 and EP2). While the southern, eastern, and western parts of the distribution range at lower latitudes will decrease as a result of future climate change (Fig. 2 EP2 and EP3 and Fig. 3 EP1 and EP2). Based on the consensus model under RCP2.6 and RCP8.5, habitat loss was estimated at 74.37% and 90.19% and new habitat gain at 33.75% and 15.08%, respectively (Table 3).

### *E. strauchi* (ERS)

BIO15 (28.18%) and BIO14 (22.59%) are the two important variables affecting the distribution of Strauch’s Desert Lacerta, respectively (Table 2). The habitat suitability map for recent climate conditions shows that the northwest, Alborz, and Kopet Dagh mountains and the northeast of Iran have 50 to 100% (especially 75–100%) suitability (Fig. 2 ERS1). Based on future climate change (especially RCP8.5), suitable habitats (especially 75–100% suitability) will be maintained in higher latitudes. While the southern margins of the distribution range, as well as the northeast sections, will decrease as a result of future climate change (Fig. 2 ERS2 and ERS3 and Fig. 3 ERS1 and ERS2). Based on the consensus model under RCP2.6 and RCP8.5, habitat loss was estimated at 25.28% and 61.09%, and new habitat gain at 0.14% and 0%, respectively (Table 3).

### *E. angramainyu* (EA)

BIO12 (39.80%) and BIO5 (31.76%) are the two important variables affecting the distribution of Angra Mainyu Leopard Gecko, respectively (Table 2). The habitat suitability map for recent climate conditions shows that the west and southwest, along with the Zagros mountains, have 50 to 100% (especially 75–100%) suitability (Fig. 2 EA1). Based on future climate change (especially RCP8.5), suitable habitats, in addition to maintaining the recent range, will expand to higher latitudes (Fig. 2 EA2 and EA3 and Fig. 3 EA1 and EA2). Expansion to higher elevations for habitats with 75–100% suitability is also apparent in the Zagros mountains (Fig. 2 EA2 and EA3). According to RCP8.5, habitat loss will occur at low elevations in the western and southern margins of the Zagros mountains (Fig. 3 EA1 and EA2). Based on the consensus model under RCP2.6 and RCP8.5, habitat loss was estimated at 0.10% and 4.61%, and new habitat gain at 30.78% and 68.58%, respectively (Table 3).

### *E. schneideri* (EUS)

BIO12 (55.41%) and BIO4 (12.61%) are the two important variables affecting the distribution of Schneider’s Long-legged Skink, respectively (Table 2). The habitat suitability map for recent climate conditions shows that most of the northwest, west, and southwest, along with the Zagros range, Alborz and Kopet Dagh mountains, the northeast, and a small portion of southeastern Iran have 50 to 100% suitability (Fig. 2 EUS1). The deserts of central and northeast Iran are devoid of EUS. Based on future climate change (especially RCP8.5), suitable habitats (especially 75–100% suitability), in addition to maintaining the recent range, will expand to higher latitudes (Fig. 2 EUS2 and EUS3 and Fig. 3 EUS1 and EUS2). Expansion to higher elevations for habitats with 75–100% suitability is also apparent in the Zagros, Alborz, and Kopet Dagh mountains (Fig. 2 EUS2 and EUS3). According to both scenarios, especially RCP8.5, habitat loss will occur on the eastern margins of the Zagros mountains, especially toward the south, as well as a small portion of the northeast and southeast (except RCP2.6) of the distribution range in the low latitudes (Fig. 3 EUS1 and EUS2). Based on the consensus model under RCP2.6 and RCP8.5, habitat loss was estimated at 9.03% and 14.04%, and new habitat gain at 53.45% and 70.92%, respectively (Table 3).

### *H. flaviviridis* (HF)

BIO1 (29.24%) and BIO15 (23.88%) are the two important variables affecting the distribution of Yellow-bellied House Gecko, respectively (Table 2). The habitat suitability map for recent climate conditions shows that the Mesopotamian plains and the coastal areas around the Strait of Hormuz and the Gulf of Oman have 50 to 100% (especially 75–100%) suitability (Fig. 2 HF1). Based on future climate change (especially RCP8.5) suitable habitats (especially 75–100% suitability) in addition to maintaining the recent range will expand to higher latitudes (Fig. 2 HF2 and HF3 and Fig. 3 HF1 and HF2). Based on the consensus model under RCP2.6 and RCP8.5, habitat loss was estimated at 1.27% and 0% and new habitat gain at 20.03% and 83.46%, respectively (Table 3).

### *H. persicus* (HP)

BIO15 (31.00%) and BIO12 (24.95%) are the two important variables affecting the distribution of Persian House Gecko, respectively (Table 2). The habitat suitability map for recent climate conditions shows that the west and southwest, along with the Zagros range, the coastal areas around the Strait of Hormuz, and the Gulf of Oman toward the southeast of Iran, have 50 to 100% (especially 75–100%) suitability (Fig. 2 HP1). Based on future climate change (especially RCP8.5), suitable habitats (especially 75–100% suitability), in addition to maintaining the recent range, will expand to higher latitudes (Fig. 2 HP2 and HP3 and Fig. 3 HP1 and HP2). Expansion to higher elevations is also apparent in the Zagros mountains (Fig. 2 HP2 and HP3). Based on the consensus model under RCP2.6 and RCP8.5, habitat loss was estimated at 1.74% and 0.41%, and new habitat gain at 11.21% and 34.24%, respectively (Table 3).

### *I. brandtii* (IB)

BIO12 (36.85%) and BIO14 (17.90%) are the two important variables affecting the distribution of Brandt’s Iranian Lacerta, respectively (Table 2). The habitat suitability map for recent climate conditions shows that the northwest to central Zagros mountains have 50 to 100% suitability. Habitats with 75 to 100% suitability are more concentrated in the northwest of Iran (Fig. 2 IB1). Based on future climate change (especially RCP8.5), suitable habitats (especially 75–100% suitability) will be maintained in higher latitudes. While the eastern, western, and southern margins of the distribution range will decrease as a result of future climate change (Fig. 2 IB2 and IB3 and Fig. 3 IB1 and IB2). Based on the consensus model under the RCP2.6 and RCP8.5 scenarios, habitat loss was estimated at 20.13% and 47.76% and new habitat gain at 0.46% and 0.12%, respectively (Table 3).

### *L. nupta* (LN)

BIO12 (36.79%) and BIO5 (21.43%) are the two important variables affecting the distribution of Large-scaled Rock Agama, respectively (Table 2). The habitat suitability map for recent climate conditions shows that the Zagros mountains, south, southeast, northeast and center of Iran have 50 to 100% suitability (Fig. 2 LN1). Habitats with 75 to 100% suitability are concentrated in the west and southwest, along with the Zagros mountains (Fig. 2 LN1). Based on future climate change (especially RCP8.5), suitable habitats (especially 50–75% suitability), in addition to maintaining the recent range, will expand to higher latitudes. Expansion to higher elevations for habitats with 50–75% suitability is also apparent in the Zagros mountains (Fig. 2 LN2 and LN3 and Fig. 3 LN1 and LN2). According to both scenarios, especially RCP8.5, habitat loss will occur on the eastern margins of the Zagros mountains, as well as the south and southeast of the distribution range in the low latitudes (Fig. 3 LN1 and LN2). Based on the consensus model under RCP2.6 and RCP8.5, habitat loss was estimated at 16.20% and 24.58% and new habitat gain at 43.13% and 73.37%, respectively (Table 3).

### *M. aspratilis* (MA)

BIO12 (61.48%) and BIO5 (12.93%) are the two important variables affecting the distribution of the Iranian Middle-toed Gecko, respectively (Table 2). The habitat suitability map for recent climate conditions shows that the west and southwest, along with the Zagros mountains, have 50 to 100% (especially 75–100%) suitability (Fig. 2 MA1). Based on future climate change (especially RCP8.5), suitable habitats, in addition to maintaining the recent range, will expand to higher latitudes (Fig. 2 MA2 and MA3 and Fig. 3 MA1 and MA2). Expansion to higher elevations for habitats with 50–100% suitability is also apparent in the Zagros mountains (Fig. 2 MA2 and MA3). According to both scenarios, especially RCP8.5, habitat loss will occur in low elevations along the western margins of the Zagros mountains as well as southern margins in low latitudes (Fig. 3 MA1 and MA2). Based on the consensus model under RCP2.6 and RCP8.5, habitat loss was estimated at 3.95% and 4.12%, and new habitat gain at 43.83% and 76.70%, respectively (Table 3).

### *M. heterocercum* (MEH)

BIO1 (25.54%) and BIO5 (19.68%) are the two important variables affecting the distribution of Blanford’s Middle-toed Gecko, respectively (Table 2). The habitat suitability map for recent climate conditions shows that the west and southwest along the Zagros mountains have 50 to 100% suitability (Fig. 2 MEH1). Habitats with 75 to 100% suitability are concentrated in the west along the Zagros mountains (Fig. 2 MEH1). Based on future climate change (especially RCP8.5), suitable habitats, in addition to maintaining the recent range, will expand to higher latitudes (Fig. 2 MEH2 and MEH3 and Fig. 3 MEH1 and MEH2). Expansion to higher elevations for habitats with 75–100% suitability is also apparent in the Zagros mountains (Fig. 2 MEH2 and MEH3). According to both scenarios, especially RCP8.5, habitat loss will occur in the western and southern margins of the Zagros mountains at low elevations and latitudes, respectively (Fig. 3 MEH1 and MEH2). Based on the consensus model under RCP2.6 and RCP8.5, habitat loss was estimated at 7.79% and 7.92% and new habitat gain at 25.07% and 48.43%, respectively (Table 3).

### *M. watsonana* (MW)

BIO12 (36.88%) and BIO1 (19.33%) are the two important variables affecting the distribution of Watson’s Sand Lizard, respectively (Table 2). The habitat suitability map for recent climate conditions shows that the southwest along the Zagros mountains, the Mesopotamian plain, all of the Iranian plateau (except the Dasht-e Kavir and Dasht-e Lut deserts), and the south of Alborz and Kopet Dagh have 50 to 100% suitability (Fig. 2 MW1). Based on future climate change (especially RCP8.5), suitable habitats, in addition to maintaining the recent range, will expand in higher latitudes, especially in Dasht-e Kavir (Fig. 2 MW2 and MW3 and Fig. 3 MW1 and MW2). Expansion to the higher elevations in the Alborz and Zagros mountains, as well as expansion to the west of Iran and the Mesopotamian plain, are also apparent (Fig. 2 MW2 and MW3). According to RCP8.5, habitat loss will occur in the southern parts of the distribution at low latitudes as well as in the northeast of the distribution range (Fig. 3 MW1 and MW2). Based on the consensus model under RCP2.6 and RCP8.5, habitat loss was estimated at 34.06% and 30.01%, and new habitat gain at 40.18% and 103.58%, respectively (Table 3).

### *M. helenae* (MIH)

BIO12 (56.24%) and BIO5 (13.40%) are the two important variables affecting the distribution of Helen’s Tiny Gecko, respectively (Table 2). The habitat suitability map for recent climate conditions shows that the west and southwest along the Zagros mountains, as well as a small portion of the Mesopotamian plain, have 50 to 100% (especially 75–100%) suitability (Fig. 2 MIH1). Based on future climate change (especially RCP8.5) suitable habitats in addition to maintaining the recent range will expand to higher latitudes (Fig. 2 MIH2 and MIH3 and Fig. 3 MIH1 and MIH2). Expansion to higher elevations for habitats with 75–100% suitability is also apparent in the Zagros mountains (Fig. 2 MIH2 and MIH3). According to RCP8.5, habitat loss will occur in the western margins of the Zagros mountains in low elevation, as well as southern margins at low latitudes (Fig. 3 MIH1 and MIH2). Based on the consensus model under RCP2.6 and RCP8.5, habitat loss was estimated at 1.85% and 11.15% and new habitat gain at 47.78% and 95.07%, respectively (Table 3).

### *M. latifi* (ML)

BIO5 (26.91%) and BIO1 (26.31%) are the two important variables affecting the distribution of Latifi’s Tiny Gecko, respectively (Table 2). The habitat suitability map for recent climate conditions shows that the western, eastern, and southern Zagros mountains and the central Iranian Plateau (except the Dasht-e Kavir and Dasht-e Lut deserts) have 50 to 100% suitability (Fig. 2 ML1). Based on future climate change (especially RCP8.5), suitable habitats will expand to the northwest and the heights of the Zagros, Alborz, and Kopeh Dagh mountains in higher latitudes (Fig. 2 ML2 and ML3 and Fig. 3 ML1 and ML2). According to both scenarios, especially RCP8.5, habitat loss will occur in the eastern and southern parts of the distribution range, especially at low latitudes (Fig. 3 ML1 and ML2). Based on the consensus model under RCP2.6 and RCP8.5, habitat loss was estimated at 58.74% and 92.84%, and new habitat gain at 60.15% and 122.23%, respectively (Table 3).

### *M. persicus* (MP)

BIO15 (50.85%) and BIO1 (29.24%) are the two important variables affecting the distribution of Persian Tiny Gecko, respectively (Table 2). The habitat suitability map for recent climate conditions shows that the southwest, south, and southeast of Iran have 50 to 100% (especially 75–100%) suitability (Fig. 2 HF1). Based on future climate change (especially RCP8.5), suitable habitats, in addition to maintaining the recent range, will expand to higher latitudes in the northern margins of the southern and southwestern ranges. Expansion onto the Mesopotamian plain is also apparent, especially in RCP8.5 (Fig. 2 MP2 and MP3 and Fig. 3 MP1 and MP2). According to RCP8.5, habitat loss will occur in the northern margins of the southeastern range (Fig. 3 MP1 and MP2). Based on the consensus model under RCP2.6 and RCP8.5, habitat loss was estimated at 9.20% and 5.23%, and new habitat gain at 2.47% and 18.31%, respectively (Table 3).

### *O. elegans* (OE)

BIO12 (39.50%) and BIO14 (17.69%) are the two important variables affecting the distribution of Elegant Snake-eyed Lizard, respectively (Table 2). The habitat suitability map for recent climate conditions shows that the northwestern, western, and southwestern parts of the country around and along the Zagros range, the Mesopotamian plain, the southern Alborz, a part of the Kopet Dagh mountains, and the southern Iranian plateau have 50 to 100% suitability (Fig. 2 OE1). Based on future climate change in the RCP2.6, suitable habitats (especially 75–100%), in addition to maintaining the recent range, will expand to the northwestern and northern regions of the high latitudes (Fig. 2 OE2 and Fig. 3 OE1). According to the RCP8.5 scenario, the western margin of the northern distribution, the western and southwestern, especially the Mesopotamian plain, the southern and eastern margins of the distribution area around and along the Zagros Mts., and the southern Iranian plateau at lower latitudes will be lost (Fig. 2 OE3 and Fig. 3 OE2). Based on the consensus model under RCP2.6 and RCP8.5, habitat loss was estimated at 33.20% and 78.89%, and new habitat gain at 66.52% and 51.85%, respectively (Table 3).

### *P. caucasia* (PC)

BIO1 (32.99%) and BIO5 (25.07%) are the two important variables affecting the distribution of Caucasian Agama, respectively (Table 2). The habitat suitability map for recent climate conditions shows that the Kopet Dagh and Alborz ranges, northwestern of the country towards the central and southwestern Zagros, as well as eastern Iran, have 50 to 100% (especially 75–100%) suitability (Fig. 2 PC1). According to RCP2.6, habitats with 75–100% suitability will be lost at low latitudes, whereas these habitats will remain at higher latitudes, especially in the Kopet Dagh, Alborz, and Zagros highlands and northwest of Iran (Fig. 2 PC2, Fig. 3 PC1). According to the RCP8.5, the habitats with 50–100% suitability will remain at higher latitudes, especially in Kopet Dagh, the Alborz highlands, northwest, and as well as a small portion of the Zagros highlands (Fig. 2 PC3). The range of species shift in the RCP8.5 scenario reveals a significant loss in the entire range of species distribution (Fig. 3 PC2). Based on the consensus model under RCP2.6 and RCP8.5, habitat loss was estimated at 59.32% and 94.67%, and new habitat gain at 9.25% and 4.03%, respectively (Table 3).

### *P. maculatus* (PM)

BIO12 (36.73%) and BIO5 (17.42%) are the two important variables affecting the distribution of Spotted Toad-headed Agama, respectively (Table 2). The habitat suitability map for recent climate conditions shows that most of the central Iranian plateau has 50 to 100% suitability (Fig. 2 PM1). Based on future climate change (especially RCP8.5), suitable habitats (especially 75 to 100% suitability), in addition to maintaining the recent range, will expand in higher latitudes (Fig. 2 PM2 and PM3 and Fig. 3 PM1 and PM2). According to both scenarios, especially RCP8.5, habitat loss will occur in the margins of the distribution range, especially in the southeastern parts at low latitudes (Fig. 3 PM1 and PM2). Based on the consensus model under RCP2.6 and RCP8.5, habitat loss was estimated at 37.58% and 35.19%, and new habitat gain at 57.18% and 94.16%, respectively (Table 3).

### *P. persicus* (PP)

BIO12 (22.31%) and BIO15 (21.77%) are the two important variables affecting the distribution of Persian Toad-headed Agama, respectively (Table 2). The habitat suitability map for recent climate conditions shows that the northwest country towards the central and small portion of the southwestern Zagros mountains, as well as the Kopet Dagh and Alborz ranges, have 50 to 100% suitability (Fig. 2 PP1). Based on future climate change, suitable habitats (especially 75 to 100% suitability), in addition to maintaining the recent range, will expand into higher latitudes (Fig. 2 PP2 and PP3 and Fig. 3 PP1 and PP2). According to both scenarios, especially RCP8.5, habitat loss will occur in the southern margins of the distribution range at low latitudes (Fig. 3 PP1 and PP2). Based on the consensus model under RCP2.6 and RCP8.5, habitat loss was estimated at 10.91% and 37.32%, and new habitat gain at 87.11% and 90.96%, respectively (Table 3).

### *P. scutellatus* (PS)

BIO12 (44.04%) and BIO1 (22.49%) are the two important variables affecting the distribution of Gray Toad-headed Agama, respectively (Table 2). The habitat suitability map for recent climate conditions shows that a wide range of the central plateau has 50 to 100% suitability (Fig. 2 PS1). In RCP8.5, habitats with 75–100% will contract as a result of future climate change, whereas habitats with 50–75% suitability will expand, especially at higher latitudes (Fig. 2 PS3). According to both scenarios, especially RCP8.5, new habitats will be gained in the Alborz, Zagros, south, and east highlands, whereas habitat loss will occur at lower elevations in the northern regions and will be restricted to low latitudes in the southern regions (Fig. 3 PS1 and PS2). Based on the consensus model under RCP2.6 and RCP8.5, habitat loss was estimated at 59.74% and 88.10%, and new habitat gain at 28.89% and 17.13%, respectively (Table 3).

### *T. caspius* (TC)

BIO1 (30.36%) and elevation (28.37%) are the two important variables affecting the distribution of Caspian Thin-toed Gecko, respectively (Table 2). The habitat suitability map for recent climate conditions shows that the west of the Caspian Sea, Hyrcanian forests, the southern range of the Alborz, east of the Alborz and Kopet Dagh mountains, east and also a small part of the central and southern Zagros highlands have 50 to 100% suitability (Fig. 2 TC1). Habitats with 75 to 100% suitability are concentrated in the west of the Caspian Sea, Hyrcanian forests, the southern range of the Alborz, and northeast of Iran (Fig. 2 TC1). Future climate change will significantly decrease the extent of habitats with the suitability of 75–100%, especially in the RCP8.5 scenario, and they will be shifted to the heights of the Alborz and Kopet Dagh ranges (Fig. 2 TC2 and TC2). According to both scenarios, especially RCP8.5, habitat loss will occur at lower elevations in the northern regions (RCP8.5) and will be restricted to low latitudes (both scenarios) in the southern regions (Fig. 3 TC1 and TC2). Based on the consensus model under RCP2.6 and RCP8.5, habitat loss was estimated at 39.90% and 79.30% and new habitat gain at 16.07% and 10.26%, respectively (Table 3).

### *T. princeps* (TP)

BIO12 (53.03%) and BIO15 (14.41%) are the two important variables affecting the distribution of Prince Lacerta, respectively (Table 2). The habitat suitability map for recent climate conditions shows that the west and southwest along the Zagros mountains, as well as the Mesopotamian plain, have 50 to 100% (especially 75–100%) suitability (Fig. 2 TP1). According to both scenarios, especially RCP8.5, future climate change will decrease the extent of habitat with 75–100% suitability, especially at lower latitudes (Fig. 2 TP2 and TP3). Under both scenarios, especially RCP8.5, habitat loss will occur at the western, southern, and eastern margins of the species distribution range in the Zagros mountains, but new habitats will be gained in the north of the distribution range and at higher latitudes (Fig. 3 TP1 and TP2). Based on the consensus model under RCP2.6 and RCP8.5, habitat loss was estimated at 14.55% and 29.83%, and new habitat gain at 2.63% and 4.15%, respectively (Table 3).

### *T. septemtaeniata* (TS)

BIO12 (57.67%) and BIO5 (16.90%) are the two important variables affecting the distribution of Southern Grass Skink, respectively (Table 2). The habitat suitability map for recent climate conditions shows that the northwest, west, and southwest, along with the Zagros mountains, the Mesopotamian plain, north, northeast, along with the Kopet Dagh ranges, and east have 50 to 100% suitability. Habitats with 75 to 100% suitability are concentrated in the middle and southern Zagros mountains (Fig. 2 TS1). Based on future climate change (especially RCP8.5), suitable habitats (especially 75–100% suitability), in addition to maintaining the recent range, will expand to higher latitudes in the northwest, north, northeast, and east of the country (Fig. 2 TS2 and TS3 and Fig. 3 TS1 and TS2). Expansion to higher elevations for habitats with 50–100% suitability is also apparent in the Zagros and Kopet Dagh mountains (Fig. 2 TS2 and TS3). Under both scenarios, especially RCP8.5, habitat loss will occur in the Mesopotamian plains and the southern margins of the Zagros mountains at lower latitudes, whereas new habitats will be gained at higher latitudes (Fig. 3 TS1 and TS2). Based on the consensus model under RCP2.6 and RCP8.5, habitat loss was estimated at 20.19% and 20.77%, and new habitat gain at 58.73% and 107.96%, respectively (Table 3).

### *T. agilis* (TA)

BIO1 (24.36%) and BIO4 (21.08%) are the two important variables affecting the distribution of Agile Ground Agama, respectively (Table 2). The habitat suitability map for recent climate conditions shows that almost all of Iran, except the northwestern part of the Zagros, has 25 to 100% suitability (Fig. 2 TA1). Based on future climate change (especially RCP8.5), suitable habitats, in addition to maintaining the recent range, will expand to higher latitudes (Fig. 2 TA2 and TA3 and Fig. 3 TA1 and TA2). Northwest of the species' distribution range at higher latitudes, especially according to the RCP8.5, may also become habitats with the suitability of 50–75% (Fig. 2 TA2 and TA3). Habitat loss is apparent in the east, south, and southeast of the species distribution range in Iran (Fig. 3 TA1 and TA2). Based on the consensus model under RCP2.6 and RCP8.5, habitat loss was estimated at 31.87% and 34.17%, and new habitat gain at 72.15% and 143.79%, respectively (Table 3).

### *T. ruderatus* (TR)

BIO12 (58.52%) and BIO5 (12.02%) are the two important variables affecting the distribution of Horny-scaled Ground Agama, respectively (Table 2). The habitat suitability map for recent climate conditions shows that the northwestern, western, and southwestern parts of the country, along with the Zagros range, Mesopotamian plain, and northeast of Iran, have 50 to 100% suitability (Fig. 2 TR1). Based on future climate change, especially RCP8.5, suitable habitats (especially 75–100%), in addition to maintaining the recent range, will expand to the northwestern regions of the high latitudes. Expansion to higher elevations for habitats with 75–100% suitability is also apparent in the Zagros mountains (Fig. 2 TR2 and TR3, Fig. 3 TR1 and TR2). Additionally, the northeastern part of the country might potentially act as a potential distribution range for the species, especially under the RCP8.5. According to the RCP8.5, the eastern and southern margins of the Zagros mountains at low latitudes and elevation, as well as the northwest of the distribution range at low elevation, will be lost (Fig. 2 TR3 and Fig. 3 TR2). Based on the consensus model under RCP2.6 and RCP8.5, habitat loss was estimated at 8.21% and 25.74%, and new habitat gain at 52.36% and 67.24%, respectively (Table 3).

### Protected area coverage

The percentage of habitat suitability and the percentage overlap of the PAs network on the habitat suitability maps for each species in recent and 2070 under the RCP8.5 scenario are provided in Table 4. All species have a small area of suitable habitat (especially ≥ 75%) within PAs, both in recent and in 2070 (Table 4). Under RCP8.5, areas of habitat with the suitability of 75–100% would decrease within PAs for 12 species, while they would increase for the remaining 18 species, though the change (decrease or increase) is not substantial (Table 4).

## Discussion

The fingerprint of climate change has been reported across a variety of taxonomic groupings that are expected to undergo elevational or poleward shifts in their geographical ranges as a result of global warming in North America, Europe, and Australia^3,5,6,36,44–48^. However, there is little knowledge about the latitudinal expansion of lizards under future climate change, especially in Asia. According to eSDM results, 83.33% of the 30 lizards studied expanded their range to the north at higher latitudes, while preserving their recent range. On the other hand, the range of 73.33% of species is restricted slightly (43.33%) or excessively (30%) along the southern margins at lower altitudes, but it will also persist or expand to higher latitudes. Following previous research on reptiles^49–51^, the findings of this study also indicate that species situated in mountainous areas (N = 19) such as the Zagros, Alborz, and Kopet Dagh may move or maintain their range at higher elevations as a result of future climate change. However, it should be highlighted that although ascending to higher elevations can provide favourable temperatures for threatened species, it can also pose challenges due to factors such as radiation, vegetation cover, and low partial pressure of oxygen (PO2)^52–54^, which are characteristics of high elevations and require further investigation for these species. According to the results of this study, annual precipitation for 16 (53.33%); annual mean temperature for 7 (23.33%); precipitation seasonality for 3 (10%); the max temperature of the warmest month for 2 (6.66%) and elevation for 1 (3.333%) of species were the most important variables influencing the Iranian lizards' distribution range (Table 2).

Reptiles have intermediate mobility^36^. Therefore, assumptions of unlimited or null dispersion under climate change are impossible, and future range shifts will probably fall in between^14^. Despite the fact that no research has been conducted on the dispersal ability of Iranian reptiles, particularly lizards, in the face of climate change, few studies in Europe can provide insight on this matter. For example, two southern European squamates, *Hierophis viridifl avus* and *Vipera aspis*, have shifted 60 km north in the last 40 years^55^. This is because warming in the colder northern ranges of species may open up new chances for colonization^14,56^. Moreno-Rueda et al. (2011) showed the mean latitude of the Spanish reptiles' ranges as they migrated northward at a rate of 0.5 km/year between 1940–1975 and 1991–2005^37^. They suggest that the rate of species migration to the north is influenced not only by dispersion ability but also by other variables such as geographic barriers and habitat distribution^37^. As a result, for the species under investigation, more research in these areas is required. The present study, which assumed an unlimited dispersion hypothesis, predicted the range loss and gain of 30 Iranian lizards by 2070, as shown in Table 3. In this study, retreat from lowlands or their southern areas was also observed for species (Figs. 2 and 3). Similar results were observed for the *Vipera berus* that retreated their distribution from the southern range in some regions of France^55^. Another example of range retraction is illustrated by field observations of many populations across the common lizard's distribution region in Europe. According to monitoring in the species' southern range, several lowland populations went extinct in 10 years, or their density was reduced by more than 50% after a warm spell^56^.

Species distribution models based on climatic factors can provide important knowledge on how species will respond to future climate change^14^. Furthermore, the findings of this study may reveal new insights into the fate of mid-latitude lizards as a result of future climate change. On the other hand, elevation can limit species ranges and has been demonstrated to have a role in explaining the distribution of species^57–60^. In this study, however, climatic factors were shown to be more significant than elevation in the distribution range of the majority of species (N = 29; Table 2), which followed previous research on reptile species richness in Iran^39,61^. Even though the results of this study shed light on how the species may respond to future climate change, it is important to acknowledge that the models in this study do not consider other factors that may contribute to lizard declines, such as anthropogenic pollution, habitat fragmentation, and loss, invasive species predation, disease, and parasitism^62^. For example, several studies have demonstrated that habitat fragmentation negatively impacts the dispersal of lizard species^63–67^. Restricted dispersal can lead to inbreeding, smaller population sizes, and loss of genetic variation^68–75^. However, there are few studies on Iranian lizards in this area, and more research is required, especially in light of climate change. On the other hand, non-climatic factors may have a major role in predicting the ranges of taxa^76,77^, and their inclusion in models, as well as feedback interactions between variables, is expected to improve future estimates of species extinction or decline^14^. Such factors include, for example, habitat management, the spatial distribution of habitats, human disturbance, and nutritional factors. Therefore, to address these complicated relationships, multi-factorial research would be necessary^78^.

The responses of closely related species to environmental conditions are generally similar, but species-specific responses have also been reported^79–85^. Depending on these two scenarios, the conservation implications may be different^79^. According to this study, climate suitability for some closely related species may be species-specific. For example, *P. maculatus* and *P. persicus*, among the three *Phrynocephalus* species evaluated in this study, will have the potential to expand their distribution range as a result of future climate change. However, the range of *P. scutellatus* may be significantly reduced (Tables 3 and 4, Fig. 3). As a result, this study suggests further investigation into phylogenetic niche conservatism and divergence among *Phrynocephalus* species, emphasizing the importance of understanding deep-time species history and speciation mechanisms before assuming common responses and conservation strategies delineation. Because it is now known that climate factors play an important role in speciation by promoting range fragmentation that leads to allopatric speciation (through niche conservatism) or promoting parapatric population divergence along climatic gradients (through niche divergence)^83^. Despite this, studies on different species of an Iranian lizard genus are rare, necessitating more research and study in this area.

Conservation organizations are being encouraged to adopt proactive efforts to reduce the effects of climate change on biodiversity^26^. Despite the need to conserve Iranian biodiversity, including lizards, from climate change, stakeholders and environmental authorities have issued no specific recommendations for the management of lizards that may be threatened. This study found that a small area of highly suitable habitat exists within the PAs (Table 4). On the other hand, this study suggests that 30% of species (n = 9) may be threatened in the future, particularly along their southern margins (Figs. 2 and 3, Table 3). Additionally, the coverage of suitable habitats (75–100%) within PAs for these species (except OE) would also decrease under future climate change (Table 4). According to the findings of this study, future climate change has resulted in a loss of suitable habitats (e.g. for AC, EP, ERS, IB, OE, PC, PS, TC, and TP) as well as habitat fragmentation (e.g. for EP, ERS, IB, PC, PS, and TC) for these species (Tables 3 and 4; Figs. 2 and 3), which can lead to a reduction in population size^86^. It should also be noted that, although future climate change may result in the expansion of suitable habitats for species (Fig. 3), these new habitats may not be protected or may be less suited than existing habitats^17,87,88^. For example, (i) changing habitat may reduce food intake because new habitats are unfamiliar or of lower quality; (ii) individuals changing social environments may encounter higher aggressiveness from nonfamiliar or nonkin individuals or may prevent the evolution of helping; (iii) individuals may face increased predation risk during the dispersal phase and early in the settling phase in all cases^89^.

Monitoring programs that track lizards' temporal and spatial changes are rare in Iran, and financing such projects should be prioritized as a research priority. Consensus over monitoring schemes and collaboration, as well as monitored species, will be required to achieve these targets. In addition, experiments on the effects of climate change should also be conducted to gain a better understanding of the mechanisms, the causal pathways involved, and nonlinear reactions to future warmer temperatures^56^. This study assessed the effectiveness of the existing PAs network and identified potential conservation areas outside the existing PAs. However, more research into human activities and the presence of natural barriers in the region is required. This new data could support the development of predictive models to define management strategies and prioritize species in Iran. In conclusion, these initial findings can contribute to improving our understanding of the ecology and biology of 30 Iranian lizards, which may be applied to future research and biomonitoring programs, as well as practical conservation actions.

## Methods

### Study area, species, and occurrence records

This study focuses on Iran, which has a total area of 1.6 × 106 km^2^ and is located in southwest Asia between the longitudes of 44° and 63° East and latitudes of 25° and 40° North (Fig. 1). The present study investigated 30 lizard species from 22 genera. These species were chosen for two reasons: (1) they had an adequate number of distribution points, and (2) their distribution range was in the west, east, north, south, center, or the entire country, allowing the response of different species across the country to be investigated under future climate change. Table 1 provides a list of these species, along with their conservation status. There are 13 species with the least concern conservation status, 12 species that are not listed, four species with data deficient, and one species that is vulnerable (Table 1). Of these, four species are endemic to Iran (Table 1). The occurrence points for these species were provided by Global Biodiversity Information Facility (GBIF, http://www.gbif.org/). To decrease the impact of spatial autocorrelation, duplicate records were removed and occurrence records with a distance of more than 1 km were employed in the analysis^90^. The number of occurrence records used for each species is listed in Table 1. The geographical coordinates of these points are illustrated in Fig. 2.

### Explanatory variables

Topography and climate are introduced as the most critical factors on reptile richness at the global and regional scales^39,91–94^. According to this, lizard's niche models were constructed for recent (1970–2000) and future (2070; the average for 2061–2080) climate change projections. Six bioclimatic variables with 30-s spatial resolution raster grids were downloaded from the WorldClimate (v 1.4) database (https://www.worldclim.org). These bioclimatic variables were annual mean temperature (BIO1 hereafter); temperature seasonality (BIO4 hereafter); the max temperature of the warmest month (BIO5 hereafter), annual precipitation (BIO12 hereafter); precipitation of driest month (BIO14 hereafter); and precipitation seasonality (BIO15 hereafter). BIO1 and BIO12 were chosen because they are the most influential factors for the richness and distribution range of reptiles in Iran^39^. The following four variables were selected because they are likely biologically significant, are weakly associated globally, and might indicate environmental features that limit distributions^95^. The elevation with the 30-s spatial resolution was also downloaded from WorldClim.

Due to uncertainty in forecasting future climate, the distribution of species was projected using averages of 14 global climate models (GCM: BCC-CSM1-1, CCSM4, CNRM-CM5, GFDL-CM3, GFDL-ESM2G, GISS-E2-R, HadGEM2-AO, HadGEM2-ES, IPSL-CM5A-LR, MIROC-ESM-CHEM, MIROC-ESM, MIROC5, MRI-CGCM3, and NorESM1-M) from the IPPC5 (CMIP5) data under two Representative Concentration Pathway (RCP) climate change scenarios: optimistic (RCP2.6 hereafter) and pessimistic (RCP8.5 hereafter).

### Ensemble species distribution modelling (eSDM)

The ensemble of species distribution models (eSDM hereafter) is a suitability-weighted average predicted by multiple algorithms and is one of the best or most powerful techniques for predicting habitat suitability, particularly in the face of future climate change^96–101^. Ensemble forecasting helps us to solve the issue of variability in forecasts produced by various modelling approaches or global circulation models^97,102,103^. For this purpose, the “biomod2” package (v 3.4.6) was used to simulate species distribution as an eSDM in the R (v 4.2.0) programming language^104^. The default settings recommended by Guisan et al. (2018) are used in this study^101,105^. The algorithms used in this study for all species were Flexible Discriminant Analysis (FDA); Random Forest (RF, n.trees = 1000), Generalized Boosted Models (GBM, n.trees = 1000, 3 Fold Cross-Validation); Generalized Linear Models (GLM, type = 'quadratic', interaction. level = 1, the stepwise procedure using Akaike Information Criterion (AIC) criteria); Classification Tree Analysis (CTA, CV.tree = 50, 5 Fold Cross-Validation); Surface Range Envelops (SRE, quant = 0.025); and Maximum Entropy (MaxEnt.Phillips, maximum iterations = 500, https://biodiversityinformatics.amnh.org/open_source/maxent/).

These models (except MaxEnt and SRE) require presence and absence data and, therefore, need a set of pseudo-absence background data samples from the landscape of the study area. Since this process involves a random procedure caused by the random selection of the pseudo-absences (possibly stratified), Guisan et al. (2018) suggested establishing several pseudo-absence data sets to avoid sampling bias, especially for a moderate or low number of pseudo-absences. According to the method of Guisan et al. (2018), this study employs random sampling throughout the study area and is repeated three times with an equal number of presence data^106–112^. For each model, 70% of the data is used to calibrate the model (training set). The Area Under Curve-Receiver Operating Characteristics (AUC hereafter) statistics, Cohen’s kappa (KAPPA hereafter), and True Skill Statistics (TSS hereafter) were used to evaluate the remaining 30% predictive capability. However, the final set is constructed with a TSS equal to or greater than 0.70^105^.

To eliminate the splitting of the total record, this process is repeated four times^105^. The TSS value ranges from -1 to + 1, + 1 means perfect agreement, and 0.60 to 0.90 means that the model performance is fair to good^113^. AUC values greater than 0.90 are considered good, those between 0.60 and 0.90 are considered average, and those below 0.60 are considered poor^114^. The importance of the variables is consistent between models that calculate the average importance of the variables used in different sets of pseudo-absences and cross-validation runs^105^.

### Species range change (SRC)

The species range change (SRC hereafter) was calculated using the "BIOMOD_RangeSiz function" for each of the 30 species, as the difference between the number of sites lost (that is, the sites where the species may not exist in the future, but currently exists) and the number of sites gained by the species (that is, the number of sites that the species may exist in the future but does not currently exist) compared with the number of sites currently occupied^115–117^.

### Protected area coverage

Arc-Map (v 10.8) was used to classify each species' habitat suitability into four categories: low (0–25%), medium (25–50%), high (50–75%), and very high (75–100%). Then, according to IUCN criteria (criteria I, II, IV, V, VI), the polygons of Iran's protected areas (PAs hereafter) were retrieved from the World Database of Protected Areas to determine the coverage of suitable habitats with designated PAs in recent and future climate scenarios (2070) under RCP8.5^118^. National and international organizations, such as the United Nations, recognize these areas as PAs that have been recognized, designated, and managed under long-term conservation objectives^119^. The following categories were selected for assessment: Strict Nature Reserve (Ia), Wilderness Area (Ib), National Park (II), National Feature (III), Habitat/Species Management Area (IV), Protected Landscape/Seascape (V), Protected Area with Sustainable Natural Resource Use (VI) (more information available at https://www.iucn.org/). This evaluation may help in the identification of new conservation areas and the development of recommendations for improving current reserve networks.

## Data Availability

Bioclimatic variables and elevation data with a 30-s spatial resolution (~ 1 km) are available in WorldClim (https://www.worldclim.org).
